# The participation of clinical pharmacists in the treatment of patients with central nervous system infection can improve the effectiveness and appropriateness of anti-infective treatments: a retrospective cohort study

**DOI:** 10.3389/fphar.2023.1226333

**Published:** 2023-09-07

**Authors:** Jie Cheng, ChuanDong Dang, Xiao Li, JianJun Wang, Xin Huang, Yan Li, XueYan Cui

**Affiliations:** ^1^ Department of Clinical Pharmacy, The First Affiliated Hospital of Shandong First Medical University and Shandong Provincial Qianfoshan Hospital, Shandong Medicine and Health Key Laboratory of Clinical Pharmacy, Jinan, China; ^2^ Department of Clinical Pharmacy, The First Affiliated Hospital of Baotou Medical College, Baotou, China; ^3^ Department of Neurosurgery, The First Affiliated Hospital of Shandong First Medical University and Shandong Provincial Qianfoshan Hospital, Shandong Medicine and Health Key Laboratory of Neurosurgery, Jinan, China

**Keywords:** antimicrobial stewardship, clinical pharmacist, neurosurgery, central nervous system infection, antibiotics

## Abstract

**Background:** Central nervous system infection (CNSI) treatment in hospital neurosurgery emphasizes the importance of optimizing antimicrobial therapy. Timely and appropriate empiric antibiotic treatment is critical for managing patients with bacterial meningitis.

**Objectives:** To evaluate the activities of clinical pharmacists in the anti-infective treatment of patients with CNSI in neurosurgery.

**Method:** A single-center retrospective cohort study was carried out from January 2021 to March 2023 at a tertiary teaching hospital in China. The study sample included a group that received pharmacy services and a group that did not. In the pharmacy services group, the anti-infective treatment plan was led and developed by pharmacists. Pharmaceutical care, including medication therapy and all CNSI treatment regimens, was administered in daily unit rounds by pharmacists. Baseline demographics, treatment outcomes, and rational use of antibiotics were compared between the two groups, and the impact of a antimicrobial stewardship (AMS) program was evaluated.

**Results:** Of the 306 patients assessed according to the inclusion and exclusion criteria, 151 patients were included, and 155 patients were excluded due to abnormal data and missing information on antibiotic costs or antimicrobial use. Eventually, 73 were included in the pharmacy services group and 78 in the group without pharmacist participation. The antibiotic use density (AUD) of the pharmacy services group decreased from 167.68 to 127.63 compared to the group without pharmacist participation. After the pharmacist services, the AUD for linezolid decreased from 9.15% to 5.23% and that for miscellaneous agents decreased from 17.91% to 6.72%. The pharmacy services group had better improvement (*p < 0.05*) and a significantly higher score for the rational use of antibiotics (*p < 0.05*) than the group without pharmacist participation.

**Conclusion:** The clinical pharmacist services evaluation results demonstrated an essential role of clinical pharmacist-led AMS programs in the effective and appropriate use of anti-infective treatments in neurosurgery with patients with CNSI.

## 1 Introduction

Central nervous system infection (CNSI) including meningitis, ventriculitis, and brain abscess, are mainly caused by postneurosurgical infection, foreign body implantation, and traumatic brain injury and are characterized by high morbidity, high mortality, and poor prognosis (Archibald and Quisling, 2013; Wang, 2021). CNSI continue to be a significant cause of mortality and morbidity worldwide. The incidence of postneurosurgical CNSI ranges from 0.3% to 25%, the incidence of CNSI caused by craniocerebral trauma is 1.4%, and the mortality rate of postneurosurgical CNSI ranges from 3% to 33% (Archibald and Quisling, 2013; Tan et al., 2015; Hernández Ortiz et al., 2018; Pan et al., 2018). According to the 2017 Infectious Diseases Society of America’s Clinical Practice Guidelines, the most common bacterial pathogens among bacterial infection in CNSI are coagulase-negative *Staphylococcus*, *Streptococcus* pneumoniae, *Staphylococcus aureus*, and *Neisseria* meningitides (Tunkel et al., 2017). The time-consuming nature and low positive rate of cerebrospinal fluid (CSF) culture, the impact of the blood‒brain barrier, and the emergence of drug-resistant bacteria increase the difficulty of antibiotic selection and the treatment of CNSI(Dorsett and Liang, 2016; Tunkel et al., 2017). Early empiric antibiotic treatment is critical for managing patients with bacterial meningitis. (Tan et al., 2015). One retrospective study showed that initial antibiotic combination therapy improved the in-hospital mortality rate among neurocritical care unit patients with nosocomial meningitis (Li et al., 2016). Timely and appropriate anti-infective treatment regimens for CNSI are essential to increase the cure rate and improve the prognosis of CNSI to reduce patient burden (Sonneville et al., 2017; Younger and Coyle, 2019; Shun-xiao et al., 2020; Li et al., 2022). Consequently, pharmacists may make some of their most important clinical contributions in treating patients with CNS infection.

In recent years, clinical pharmacists have received increasing attention worldwide, especially during the COVID-19 pandemic (Gu et al., 2023). Studies have demonstrated the beneficial effects of the participation of clinical pharmacists in treating patients with chronic illnesses such as diabetes, atrial fibrillation, and cardiovascular disorders (Skelton and Binaso, 2012; Falamić et al., 2018; Warden et al., 2019). The Infectious Diseases Society of America guidelines highlight the importance of clinical pharmacists on the antimicrobial stewardship teams (Dellit et al., 2007). The Chinese Hospital Association and the American College of Clinical Pharmacy have established standards for clinical pharmacists’ level of clinical pharmacy services and the specialty training required for these positions (Dellit et al., 2007; China Hospital Association Clinical Pharmacist Training Expert Steering Committee, 2008; Saseen et al., 2017). Antimicrobial stewardship (AMS) programs were created to improve infection regimens and minimize infection-related morbidity and death (Barlam et al., 2016; Álvarez-Lerma et al., 2018). Evidence shows that pharmacists significantly impact multiple clinical and economic outcomes. For example, pharmacists in the discharge process benefit neurosurgical patients by reducing the number of discrepancies and providing an additional layer of safety to reduce medication errors and prevent adverse events (Greenwood et al., 2023). Another retrospective cohort study found that the presence of clinical pharmacists on a neurosurgery team considerably reduced hospital stays, readmission rates, and pharmacy expenses. Their involvement in a neurosurgical team should be considered a standard of care (Weant et al., 2009). Clinical pharmacists join the neurosurgery team to identify prescribing errors, monitor or modify doses, identify adverse effects associated with multiple drug combinations, optimize antibiotic use, and provide clinical outcomes to reduce the burden of patient care (Bourne and Dorward, 2011; Gallagher et al., 2014). Therefore, in this study, we aimed to investigate the benefits of clinical pharmacy services for patients with CNSI. We aimed to assess the effect of a clinical pharmacists services on the effectiveness and appropriateness of anti-infection treatments in CNSI patients.

## 2 Methods

### 2.1 Study design

This single-center retrospective cohort study was conducted at the First Affiliated Hospital of Shandong First Medical University and Shandong Provincial Qianfoshan Hospital (a 4000-bed tertiary care hospital) in Shandong Province, China. This study was conducted with two neurosurgical group and retrospective collecting patient information with CNSI between 2021.1.1 and 2023.3.1. The patients include two groups: the pharmacy services group, which received a routine pharmacist services during hospitalization, and group without pharmacist participation.

The study was approved by The Ethics Committee of the First Affiliated Hospital of Shandong First Medical University and Shandong Provincial Qianfoshan Hospital (approval number: 2023S357). It was conducted following the Helsinki Declaration guidelines, and informed consent was not required due to retrospective and diagnostic data collection.

### 2.2 Study participants

#### 2.2.1 Inclusion criteria

This study included patients with CNSI diagnosed in the neurosurgery department of the hospital. The diagnosis of the infection required patients to meet two or more of the following criteria (Horan et al., 2008; Archibald and Quisling, 2013): 1) isolation of pathogens from CSF; 2) at least one of the following signs with no other recognized cause: fever (>38 °C), headache, stiff neck, meningeal signs, cranial nerve signs, changing level of consciousness, or confusion; and 3) the following abnormalities on the CSF test: corrected (Greenberg et al., 2008) white blood cell count >5 × 10^6^ cells/L, protein concentration >45 mg/L, and/or CSF glucose <50 mg/dL.

#### 2.2.2 Exclusion criteria

Patients who met any of the following criteria were excluded (Gu et al., 2023): 1) patients were not using antimicrobials on admission, or antimicrobial information was missing; 2) laboratory tests showed extremely abnormal values, such as alanine aminotransferase or aspartate aminotransferase >1,000; 3) the number of days of hospitalization was <1 day or >30 days; or 4) patients had incomplete medical history information.

### 2.3 Pharmaceutical service process

#### 2.3.1 Pharmacy services group

The clinical pharmacist, who had completed specialized training and had over 10 years of hospital pharmacy experience in the neurology department, was assigned to the one neurosurgical group to ensure the consistency and quality of the treatments. In the pharmacy services group, the pharmacist implemented clinical pharmacy services by carrying out the following steps. 1) The pharmacist spent at least 20 h per week with neurosurgery physicians participating in daily unit rounds. The pharmacist focused on medication therapy management, including medical order review, patient monitoring, dynamic treatment regimens (DTRs), etc. 2) The pharmacist was involved in the development of all CNSI treatment regimens, including therapeutic indications, drug selection, dose, route of administration, and period of treatment, etc. The pharmacist adjusted the dosage based on the patient’s liver and kidney function. 3) The AMS program was implemented if the pharmacist had different opinions after revisions. 4) The adjustment plan was presented to the senior physician and an expert in neurosurgery. 5) Pharmaceutical monitoring and the adjustment treatment plans were implemented based on the treatment outcomes and safety.

#### 2.3.2 Group without pharmacist participation

In the other neurosurgical group, the treatment involved the doctor’s routine diagnosis and treatment, and pharmacists did not participate in the therapeutic schedule.

### 2.4 Outcomes and measurement

The primary outcome was clinical effectiveness, which was categorized as follows (Tunkel et al., 2004; Archibald and Quisling, 2013): 1) Cured: resolution of symptoms, negative CSF cultures, and a regular CSF examination (WBC count: <5*10^6^ cells/L, protein concentration: <45 mg/L, and/or CSF glucose: >50 mg/dL); 2) Improved: negative CSF cultures and partial resolution of clinical signs and symptoms of infection, or CSF examination improved but did not return to normal. 3) Invalid: Positive or negative CSF cultures and symptoms of infection and worsening of CSF indices compared to baseline. The secondary outcome was the appropriateness of medication, which was evaluated based on an antibiotic appropriateness assessment score. We used the 5R principles of rational drug use as our evaluation metric and constructed an analytic hierarchy process (AHP) evaluation system (Saaty, 1990; Mobinizadeh et al., 2016; Li et al., 2017). Two clinical pharmacy experts skilled in anti-infective treatment reviewed the medical records of the two groups of inpatients. They used a scoring system to assess the appropriateness of the use of antibiotics to treat CNSI. If the anti-infective treatment specialist identified inappropriate antibiotic use, the item was scored and multiplied by the appropriate weight (Table 1.) determined by the AHP to produce a final score.

**TABLE 1:** The indicators and weight of each indicator.

Items	Weight
Indication	0.42
Selection	0.26
Dosage	0.16
Route of administration	0.10
Period of treatment	0.06

Other outcomes included antibiotic consumption, medical costs, and length of hospital stay (LOS). The WHO confirmed the defined daily dose (DDD) and antibiotic use density (AUD) for antibiotics (WHO, 2022). In addition, we collected the AUD (%) and AUD decrease (%) as indicators to assess the impact of pharmacy services on antibiotic use. The cost of antibiotics, the total cost of drugs, and the total cost of hospitalization were collected. The cost of antibiotics refers to the price of antibiotics administered to the patient to treat the CNSI during hospitalization. Medical service efficiency was measured by the LOS, which was calculated by subtracting the admission date from the discharge date. In this study, a cost-effectiveness analysis (CEA) was used to assess the relationship between the cost and effectiveness of the two groups. The long-term clinical and economic outcomes of pharmacy services were calculated using the incremental cost-effectiveness ratio (ICER). The ICER calculated the difference in hospitalization costs between the pharmacy services group and the group without pharmacist participation, divided by the difference in treatment improved rate between the two groups (Tang et al., 2019). In terms of the cost-effectiveness threshold, WHO defines a health intervention as highly cost-effective if it costs less than the gross domestic product (GDP) *per capita* of a given country, and as cost-effective if it costs less than three times the GDP *per capita*. The *per capita* GDP stratified for China was $12,741.11 in 2022 (China National Bureau of Statistics, 2023). For the cost of medical, the costs used in this study were prior to Medicare reimbursement. The State Administration of Foreign Exchange in China monitored the exchange rate between the RMB and the US dollar. On 2022, the average rate was US $100 = RBM 672.61 Yuan.

### 2.5 Data collection

The following were obtained from the Hospital Information System (HIS) and laboratory Information Management System (LIS) for each inpatient. 1) General demographic attributes, including age and sex, weight, LOS, surgery and placement of invasive devices, were collected. 2) Pathogens in the CSF and the antimicrobial susceptibility results were assessed. 3) Laboratory test results were obtained, including glucose level, protein level, chlorine level, leukocyte count, and erythrocyte count in the CSF; white blood cell count, procalcitonin (PCT) value, and the levels of liver function indicators, including alanine aminotransferase (ALT), aspartate aminotransferase (AST), and total bilirubin (TBIL); and creatinine levels. The creatinine clearance rate (CrCL) was estimated by the Cockcroft-Gault method. We collected laboratory test results before and twice after the anti-infective treatment. 4) Antibiotic use, including the medication name, dosage unit, package, defined daily dose (DDD), administration route, and the time of issuing and stopping medical orders, were assessed. 5) Finally, antibiotic treatment costs were determined.

### 2.6. Statistical analysis

Descriptive statistics were used to characterize the study group. The mean and standard deviation, median (interquartile range, IQR), and number (percentage) were used to depict normally distributed, nonnormally distributed continuous, and categorical data, respectively. Normally distributed quantitative variables were analyzed for differences between groups using Student’s t-test, and nonnormally distributed data were analyzed using the Mann‒Whitney rank sum test. The qualitative variables were compared using the chi-square test or Fisher’s exact test. *p < 0.05* was considered statistically significant. All comparisons were based on two-tailed tests. SPSS Statistics v.26.0 (IBM Corp., NY, USA) was used for all statistical analyses.

## 3 Results

### 3.1 Demographic characteristics

**FIGURE 1:**
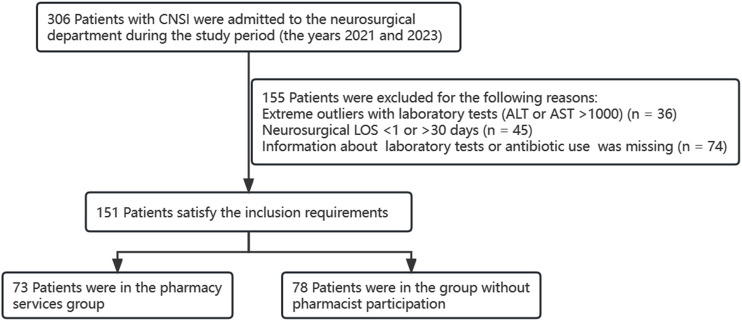
Diagram of the patient assessment and inclusion process.

A total of 306 patients with CNSI were admitted to the neurosurgical department during the study period (2021 and 2023). According to the inclusion and exclusion criteria, 151 patients were included, and 155 patients were eliminated because of abnormal data and missing information on antibiotic costs or antimicrobial usage (Figure 1.). The initial analysis included 78 patients in the group without pharmacist participation and 73 patients in the pharmacy services group. Baseline characteristics for the group without pharmacist participation and pharmacy services group are shown in Table 2. Sex, age, weight, PCT, CrCL, ALT, and creatinine were selected as baseline indicators, and the above indicators did not differ between the two groups (*p > 0.05*).

**TABLE 2:** Baseline characteristics of study patients.

	Group without pharmacist participation (N = 78)	Pharmacy-services group (N = 73)	*p*
Age, mean ± SD	51.85 ± 16.712	55.44 ± 17.197	0.195
Male n (%)	47 (51.1%)	45 (48.9%)	0.861
Female n (%)	31 (52.5%)	28 (47.5%)	
Weight, median (IQR)	50 (15.3)	60 (20.0)	0.288
PCT, median (IQR)	0.064 (0.248)	0.054 (0.147)	0.74
ALT, median (IQR)	17.6 (23.7)	20.9 (20.5)	0.12
CrCL, median (IQR)	116.46 (74.43)	102.27 (57.91)	0.119
Creatinine, median (IQR)	50.5 (24.0)	55 (22.2)	0.108

IQR, interquartile range; PCT, procalcitonin; ALT, alanine aminotransferase; CrCL, Creatinine clearance rate.

### 3.2 Quantitative descriptions of pharmacist recommendations

A total of 420 pharmacist recommendations were identified, with 399 (95.00%) reaching an agreement between the clinical pharmacist and the neurosurgery physicians. In this study, neurosurgical pharmacists achieve the AMS program primarily through the following core services, anti-infective drug selection (146/420, 34.76%) with an acceptance rate of 94.52%, anti-infective dose recommendations (103/420, 24.52%) with an acceptance rate of 95.15%, anti-infective duration recommendations (73/420, 17.38%) with an acceptance rate of 95.89%, anti-infective route recommendations (18/420, 4.29%) with an acceptance rate of 95.00%.

### 3.3 Clinical effectiveness

As shown in Table 3, the treatment outcome showed that 6 (8.2%) patients in the pharmacy services group were cured, while 49 (67.1%) were improved. Moreover, among the 78 patients in the group without pharmacist participation, 3 (3.8%) and 39 (50%) were determined to be cured and improved, respectively, and no significant difference was detected in the cure rates between the two groups (*p > 0.05*). However, the pharmacy services group had a better improvement rate than the group without pharmacist participation (*p < 0.05*). In addition, the LOS values were not significantly different between the two groups (*p > 0.05*).

**TABLE 3:** Outcomes of patients with CNSIs in the Group without pharmacist participation and pharmacy-services groups.

		Group without pharmacist participation	Pharmacy-services group	*p*
LOS, median (IQR)		18.5 (12.75.27.25)	22 (15.29)	0.11
Antibiotic costs ($), median (IQR)		1965.35 (415.32,4044.42)	2098.10 (376.29,3827.67)	0.647
Hospitalization costs ($), median (IQR)		9521.96 (4878.79,15,989.60)	16,532.85 (10,956.78,25,528.18)	<0.05
Drug costs ($), median (IQR)		4864.27 (2363.22,9474.17)	7213.33 (4130.99,11,916.24)	<0.05
ICER($)		409.99		-
Combination antibiotic	Single	19 (24.4%)	20 (27.4)	>0.05
	Double	58 (74.4%)	51 (69.9)	>0.05
	Trible	1 (1.3%)	2 (2.7%)	>0.05
Antibiotics switched	No switched	39 (50%)	62 (84.9%)	<0.05
	Switched	39 (50%)	11 (15.1%)	<0.05
DDDs, median (IQR)		32.25 (16.5.45.0)	23.0 (9.0.42.0)	0.213
Treatment outcome	cured	3 (3.8%)	6 (8.2%)	>0.05
	Improved	39 (50%)	49 (67.1%)	<0.05
	Invalid	36 (46.2%)	18 (24.7%)	<0.05
Rational use of antibiotics score, median (IQR)		0.74 (0.74.0.84)	1.00 (1.00.1.00)	<0.05

IQR, interquartile range; LOS, length of stay.

### 3.4 Appropriateness of medication

The pharmacy services group had a significantly higher score for the rational use of antibiotics than the group without pharmacist participation (*p < 0.05*), which is shown in Table 3. Analysis of combination therapy showed that no significant differences were found between the two groups for single, double or triple combinations. A study of antibiotic conversion rates showed that the switch rate of the antibiotic regimen in the pharmacy services group was much lower than that in the group without pharmacist participation (*p < 0.05*).

### 3.5 Other outcomes

We were interested in the impact of pharmacist services on antibiotic use in neurosurgical patients. The pharmacist services reduced the AUD of all antibiotics used in neurosurgery from 167.68 to 127.63. According to Table 4, the AUD proportions declined for linezolid (9.15% vs. 3.92%), miscellaneous agents (17.91% vs. 6.72%), and polymyxin B (2.82% vs. 1.06%). The AUD proportions increased by 5.37%, 3.82%, and 8.99% for vancomycin (from 21.91% to 27.28%), meropenem (from 41.02% to 44.84%), and ceftriaxone (from 7.19% to 16.17%), respectively. The pharmacist services did not significantly impact antibiotic co-administration but reduced the rate of antibiotic switches. In addition, there were no significant differences between the two groups in other indicators, such as antibiotic costs (*p = 0.647*) or DDDs (*p = 0.213*). Although the drug and hospitalization costs in the pharmacy-services group increased (*p < 0.05*), the pharmacy-services group had an ICER less than the Chinese threshold ($409.99 vs. $12,741.11).

**TABLE 4:** Effect of pharmacist services on the type and amount of antibiotics used.

	Group without pharmacist participation		Pharmacy-services group		Descender (%)
	AUD	AUD% (%)	AUD	AUD% (%)	
Total	167.68	100	127.63	100	
Vancomycin (J01XA01)	36.74	21.91	34.82	27.28	−5.37%
Meropenem (J01DH02)	68.79	41.02	57.23	44.84	−3.82%
linezolid (J01XX08)	15.35	9.15	5.01	3.92	5.23%
Ceftriaxone (J01DD04)	12.05	7.19	20.64	16.17	−8.99%
polymyxin B (J01XB02)	4.73	2.82	1.35	1.06	1.76%
miscellaneous	30.03	17.91	8.58	6.72	11.19%

Miscellaneous, antibiotics other than those already listed in the table.

## 4 Discussion

### 4.1 Statement of key findings

The National Health Commission of China issued the strictest regulation on antimicrobial drugs in 2011, as the number of antibiotic-resistant bacteria has become a severe problem in China (Hu et al., 2016). The Antimicrobial Stewardship (AMS) Program was created to solve these issues. The AMS was designed to optimize the treatment of infection, reduce infection-related morbidity and mortality, limit the emergence of multidrug-resistant organisms (MDROs), and reduce unnecessary antimicrobial use (Álvarez-Lerma et al., 2018). The AMS indicates that pharmacists have a responsibility to play a prominent role in antimicrobial stewardship, infection prevention and control programs in healthcare systems. Core elements of effective antimicrobial stewardship programs typically include hospital leadership commitment, the appointment of a pharmacist to lead the implementation of antimicrobial stewardship interventions, monitoring of antimicrobial prescribing, regular reporting of information on antimicrobial use and resistance to prescribers, and education on rational use of medicines (CDC, 2019). Pharmacists participate in antimicrobial stewardship, infection prevention and control efforts through clinical efforts focused on the appropriate use of antimicrobial agents and through membership in relevant multidisciplinary workgroups and committees within the healthcare system (Saseen et al., 2017; Lai et al., 2022). Currently, pharmacists in China mainly participate in the daily ward rounds in neurosurgery units and review medication regimens (Zhang et al., 2020b; Zhang et al., 2021; Wang et al., 2022; Gu et al., 2023). The involvement of pharmacists in patient medical treatment and monitoring is the ideal approach in neurosurgery units because of the complexity of CNSI, the severity of these diseases, and the difficulty of drug selection. In this study, a pharmacist worked in a neurosurgery unit reviewing prescriptions for patients with CNSI and discussing treatment issues with the surgeon as needed. The pharmacist engaged in medical treatment discussions and exchange of ideas. Ultimately, pharmacists use treatment regimens optimized for infected patients according to professional guidelines or treatment criteria.

In this study, we assessed the effects of the participation of clinical pharmacists in the treatment of patients with CNSI on the effectiveness of antibiotic prescriptions. The results of our study showed that the pharmacy services group had a significantly higher rate of treatment improvement and efficiency than the group without pharmacist participation (*p* < 0.05). A meta-analysis on cardiovascular disease showed a similar finding: pharmacist services provides a wide range of benefits in cardiovascular disease management, from controlling risk factors to improving medication adherence and, in some settings, reducing morbidity and mortality (Rattanavipanon et al., 2022). No significant effect of the pharmacist services on the cure rate was observed in this study, partly because CNSI have a long course of treatment, making it difficult to achieve the desired treatment outcome during hospitalization, and partly because of our strict cure criteria. The criteria for cure in this study were based on diagnostic criteria, and there is no clear literature support for the criteria for the cure of CNSI.

The AHP approach can be used for decision analysis and calculation in the healthcare system (Khan et al., 2022; Kim et al., 2022). With the help of the AHP, pharmacists can objectively and comprehensively evaluate medicine, which is convenient for identifying problems and intervening in time, thereby promoting the appropriate use of treatment (Xu et al., 2022b). We evaluated the appropriateness of antibiotic use choices against guidelines and constructed an AHP weighting system to quantify the rational use of antibiotics (Tunkel et al., 2004; Tunkel et al., 2008; Tunkel et al., 2017). We found a significant difference in the rational antibiotic use scores between the two groups, indicating that pharmacist services effectively improved the appropriateness of antibiotic use. This finding also shows that the standardization of physician prescribing still needs to be improved. A retrospective study found similar results: AMS services were associated with significant reductions in antibiotic use, antibiotic costs, and inappropriate antibiotic prescribing (Xu et al., 2022a). By analyzing the intensity of antibiotic use, we found that pharmacist services were effective in reducing the intensity of antibiotic use, a view supported by previous studies (Jourdan et al., 2018). In addition, regarding medication adherence, we found that after the pharmacist’s services, there was a significant reduction in the AUD of linezolid and miscellaneous agents. At the same time, the use of vancomycin, ceftriaxone, and meropenem in combination therapy increased. Selecting an appropriate antibiotic regimen for CNSI is critical to treatment success. Usually, vancomycin plus an anti-pseudomonal beta-lactam (such as cefepime, ceftazidime, or meropenem) is recommended as empiric therapy for healthcare-associated ventriculitis and meningitis; the choice of empiric beta-lactam agent should be based on local *in vitro* susceptibility patterns. For the treatment of patients with CNSI caused by staphylococci in whom vancomycin cannot be used, linezolid is recommended (Tunkel et al., 2017). Pharmacists ensuring that the number and types of antimicrobial agents provided in the treatment plan available are appropriate for the patient population served is one of the most critical responsibilities in the AMS program (Lai et al., 2022). Our results indicate that clinical pharmacists play a key role in the rational use of antimicrobial agents, which is consistent with other research (Gu et al., 2023). In addition, the rate of antibiotic switching also reflects the appropriateness of the choice of treatment regimen; frequent changes and combinations of antibiotics also mean that physicians are concerned about treatment failure and are not confident in their choice of antibiotics or that the treatment regimen is not the standard guideline-recommended regimen (Livorsi et al., 2015; Gebretekle et al., 2018). Our study found that the pharmacist services group had a significantly reduced rate of antibiotic switching, suggesting that pharmacists play a facilitating role in selecting an appropriate initial regimen. Pharmacist services ensured the regularity of physician prescribing behavior, the continuity of the initial treatment regimen, and increased physician confidence in the antibiotic regimen (Woit et al., 2020).

Most of the current literature (Weant et al., 2009) suggests that pharmacist services effectively reduce antibiotic costs, but our study did not find a significant difference between the two groups. Although the pharmacy services effectively reduced the total AUD, there was no significant reduction in drug costs due to the different prices of different antibiotics. Although the hospitalization costs of patients with pharmacist services were higher than the group without pharmacist involvement, the rate of improvement was higher in the pharmacist services group. The results of the CEA then showed that the pharmacy services group was highly cost-effective and that the increased costs were acceptable. Similarly, a study conducted in Thailand found that pharmacist intervention in the healthcare team to participate in the treatment plan is cost-effective and can prolong patients’ lives and improve their quality of life (Arunmanakul et al., 2022). From the hospital’s perspective, pharmacy services have not reduced direct healthcare costs. Still, it presented savings for forecast costs related to preventable morbidities, measuring an excellent cost-benefit ratio for the public health sector (Cazarim et al., 2020). The pharmacist services also did not significantly reduce the LOS, which is inconsistent with the literature (Nielsen et al., 2017). The LOS is primarily influenced by the treatment characteristics of CNSI, which are difficult to cure and take a long time to treat (Dorsett and Liang, 2016). Multiple factors contribute to the discrepancy in hospitalization costs, such as the price of guideline-recommended vancomycin is much higher than that of linezolid in China, which may be the main reason.

The results of this study show that a clinical pharmacist-led antibiotic stewardship program successfully promoted the rational use of antibiotics and improved treatment outcomes. In order to improve the rational use of medication and therapeutic outcomes in the clinic, there is a need to increase the number of clinical pharmacists and improve the competence of clinical pharmacists. So, health systems need to increase the number of clinical pharmacists and provide standardized training, etc. From a clinical pharmacist’s perspective, a qualified clinical pharmacist should engage in clinical practice, medication therapy management, patient monitoring, etc.

### 4.2 Study strengths and limitations

Our study is an assessment project led by clinical pharmacists on neurosurgical anti-infective treatments to assess their effectiveness and appropriateness. Clinical pharmacists play an essential role in developing patient anti-infection protocols and in reducing antibiotic resistance rates. Clinical pharmacists providing pharmaceutical care to neurocritical care patients improve patient care and reduce clinical risk (Bourne and Dorward, 2011). A recent systematic review showed that pharmacist services could significantly improve the acceptability and effectiveness of treatment for infectious diseases (Zhang et al., 2020a). Pharmacists are playing an increasingly important role in clinical practice, and thanks to the clinical pharmacist training program launched in China, pharmacists are equipped with professional drug knowledge and clinical experience through professional training and play a role in bridging the gap between doctors and patients and drugs, providing personalized anti-infection programs based on understanding patients’ conditions, reviewing doctors’ prescriptions to correct medication errors, and playing a greater role in patient monitoring than busy doctors and nurses.

Compared with previous studies (Weant et al., 2009), our study focused more on evaluating the therapeutic efficacy and appropriateness of anti-infective regimens after pharmacist services. The pharmacist services model was based on the AMS strategy, and the effectiveness of the services was ensured by standardized processes, clear delineation, and practical implementation methods. Our findings suggest the high value and clinical utility of participation in a clinical pharmacist service for patients with neurosurgical CNSI in general hospitals.

This study has several limitations. First, this was a single-center study with a relatively small sample size. Each medical setting has different patterns of collaboration between clinical pharmacists and neurosurgeons and different work priorities, resulting in different roles. Second, the impact of clinical outcomes such as long-term survival rates, reinfection rates, and resistance rates could not be estimated due to the need to collect such data, and we were unable to assess the long-term effects of the AMS Program due to the short duration of the study.

## 5 Conclusion

This study demonstrates that the participation of clinical pharmacists in the neurosurgery department of a general hospital can increase the rational use of antibiotics, improve treatment outcomes, and reduce the intensity of antibiotic use. Nevertheless, such participation has failed to reduce the antibiotic costs of patients and decrease the length of hospital stays. These results need to be confirmed in further studies.

$$DDDs=\frac{accumulated\;drug\;use\left(g\right)}{defined\;daily\;dose}$$

$$AUD=\frac{DDDs\;of\;specific\;antibiotic\;types}{number\;of\;days\;spent\;in\;bed\;\left(day\right)}\times 100$$

$$AUD\;\left(\%\right)=\frac{AUD\;of\;specific\;antibiotic\;types}{total\;AUD\;of\;all\;antibiotic\;types}\times 100\%$$

$$AUD\;descender\;\left(\%\right)=AUD\%\;in\;group\;without\;pharmacist\;participation-AUD\%\;in\;pharmacy\;services\;group$$

## Data Availability

The raw data supporting the conclusion of this article will be made available by the authors, without undue reservation.
